# Alleviative Effect of Geniposide on Lipopolysaccharide-Stimulated Macrophages via Calcium Pathway

**DOI:** 10.3390/ijms25031728

**Published:** 2024-01-31

**Authors:** Hyun-Ju Kim, Wansu Park

**Affiliations:** Department of Pathology, College of Korean Medicine, Gachon University, Seongnam 13120, Republic of Korea

**Keywords:** geniposide, lipopolysaccharide, inflammation, macrophages, cytokine, calcium, P38 MAPK

## Abstract

In this study, we investigated how geniposide (a bioactive ingredient of gardenia fruit) acts on lipopolysaccharide (LPS)-stimulated macrophages. Griess reagent assay, Fluo-4 calcium assay, dihydrorhodamine 123 assay, multiplex cytokine assay, quantitative RT-PCR, and flow cytometry assay were used for this study. Data showed that geniposide at concentrations of 10, 25, and 50 μM reduced significantly the levels of nitric oxide, intracellular Ca^2+^, and hydrogen peroxide in LPS-activated RAW 264.7. Multiplex cytokine assay showed that geniposide at concentrations of 10, 25, and 50 μM meaningfully suppressed levels of IL-6, G-CSF, MCP-1, and MIP-1α in RAW 264.7 provoked by LPS; additionally, geniposide at concentrations of 25 and 50 μM meaningfully suppressed the levels of TNF-α, IP-10, GM-CSF, and MIP-1β. Flow cytometry assay showed that geniposide reduces significantly the level of activated P38 MAPK in RAW 264.7 provoked by LPS. Geniposide meaningfully suppressed LPS-induced transcription of inflammatory target genes, such as *Chop*, *Jak2*, *Fas*, *c-Jun*, *c-Fos*, *Stat3*, *Nos2*, *Ptgs2*, *Gadd34*, *Asc*, *Xbp1*, *Nlrp3*, and *Par-2*. Taken together, geniposide exerts alleviative effects in LPS-stimulated macrophages via the calcium pathway.

## 1. Introduction

Immunity is an essential process for health and human life. Innate immunity protects the human body from invading microbial pathogens (i.e., bacteria). Inflammation is an early host defense response to pathogenic infections [1]. But then, uncontrolled inflammation is dangerous to host survival [2], and excessive inflammation sometimes leads to not only acute inflammatory diseases but also chronic inflammatory disorders [3]. Acute inflammation is caused by neutrophils and monocytes that immediately respond to bacterial pathogens or injured tissues and is mediated by vasoactive amines and eicosanoids. Acute inflammation lasts only a few days and then resolves but sometimes develops into abscess formation or chronic inflammation. Chronic inflammation is caused by acute inflammation that persists due to non-degradable pathogens, viral infection, or autoimmune reactions and involves monocytes, macrophages, lymphocytes, plasma cells, and fibroblasts. It is mediated by interferons, cytokines, growth factors, chemokines, reactive oxygen species, and hydrolytic enzymes. Chronic inflammation lasts for months or sometimes years, causing tissue destruction, fibrosis, and necrosis, and can lead to body pain, myalgia, chronic fatigue, insomnia, depression, gastrointestinal complications, weight loss (or gain), atherosclerosis, allergy, and cancers. As macrophages play an important role not only in acute inflammation but also in chronic inflammation, this study established them as an in vitro model of infectious agent-induced cellular inflammation. P38 MAPK controls macrophage plasticity by inducing endoplasmic reticulum stress (ERS) and glucose intolerance in infected hosts [4]. It is well known that cytokines and chemokines may contribute to the exacerbation of inflammatory diseases such as atherosclerosis [5,6]. Thus, the regulation of inflammation has become increasingly important for treating inflammatory diseases [7]. Representative cytokines associated with inflammatory diseases include interleukin-6 (IL-6). Not only cytokines but also nitric oxide (NO) are important factors involved in the inflammatory response process of macrophages [8]. Tumor necrosis factor-α (TNF-α) is also an important cytokine associated with endotoxemia [9]. Lipopolysaccharide (LPS, endotoxin) can stimulate immune cells like macrophages, which produce cytokines and hydrogen peroxide (H_2_O_2_). Monocyte chemoattractant protein-1 (MCP-1) is also one of the increased chemokines in endotoxemia. Excessive cytokines in infectious diseases may have a serious outcome in patients. Thus, LPS-induced macrophages are regarded as being useful for studying the anti-inflammatory effect of potential therapeutic agents. TNF-α-converting enzyme (TACE) is a sheddase responsible for cleaving membrane TNF, and reactive oxygen species (ROS) mediate LPS-induced upregulation of TACE activity via P38 MAPK activation in monocytes [10]. Many studies have reported that ERS-related pathways, including C/EBP homologous protein (CHOP) and protease-activated receptor-2 (PAR-2), might be upregulated through P38 MAPK phosphorylation, which is related to growth arrest and DNA damage-inducible gene 34 (GADD34) [11,12,13,14]. CHOP activation is also linked to X-box-binding protein 1 (XBP1). Recently, macrophage inflammatory proteins (MIPs) have been recognized as important in the amplification process of inflammatory phenomena. Interferon-inducible protein (IP)-10 is also increased during inflammation. In addition, it is known that not only cytokines and chemokines but also growth factors, such as increased blood levels in inflammatory diseases, worsen the condition.

**Figure 1:**
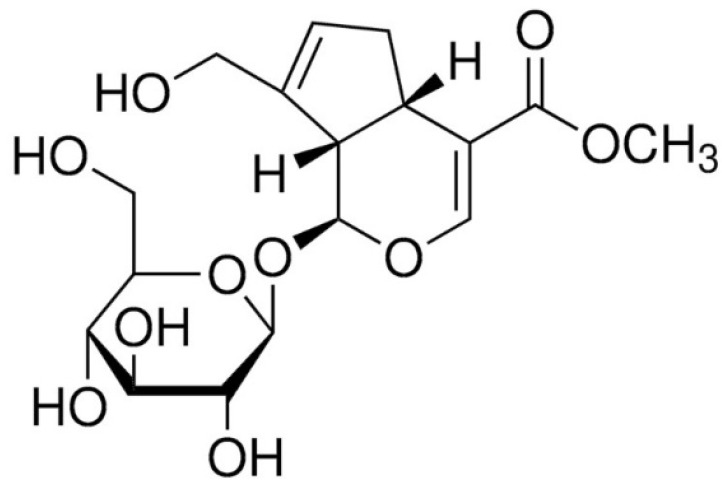
The structural formula of geniposide.

Geniposide (Figure 1) is a major ingredient in the herb gardenia [13]. The herb gardenia, a fruit of *Gardenia jasminoides*, is widely used in Asian countries such as Korea, China, India, Japan, Vietnam, Taiwan, and Myanmar to treat inflammatory disorders [15,16,17]. Geniposide is known to suppress inducible nitric oxide synthase in macrophages induced by LPS as well as cyclooxygenase-2 [16]. Cyclooxygenase-2 is also reduced by geniposide in macrophages. Cheng et al. suggested geniposide inhibits cytokines levels in RAW 264.7 induced by LPS as well as reduces the plaque size and serum TNF-α level in ApoE^−/−^ mice via microRNA-101/mitogen-activated protein kinase phosphatase-1/P38 signaling [18]. Geniposide was reported to reduce atherosclerotic plaques in rabbits via the MAPK pathway [19]. Despite such studies, the effects of geniposide on ERS-related cytokine production of LPS-provoked macrophages are still unknown. In a previous study, we reported that natural products alleviate the inflammatory response of macrophages caused by LPS through the calcium-CHOP signaling pathway [20]. However, we have not yet reported the effect of geniposide on the inflammatory response of macrophages

The main purpose of this study was to determine the effect geniposide has on the inflammatory response of macrophages caused by lipopolysaccharide and, if it exhibits anti-inflammatory effects, the detailed signaling mechanisms (such as ERS-related p38 MAPK) involved. Effects of geniposide on cytokines, H_2_O_2_, and NO in LPS-provoked RAW 264.7. In addition, quantitative PCR was carried out to measure mRNA expression of ERS-related genes such as *Chop*, Janus kinase 2 (*Jak2*), first apoptosis signal receptor (*Fas*), *c-Jun*, *c-Fos*, Signal Transducer and Activator of Transcription (*Stat*)-1, *Stat-3*, nitric oxide synthase 2 (*Nos2*), cyclooxygenase-2 (*Ptgs2*), *Gadd34*, Apoptosis-associated speck-like protein containing a caspase recruitment domain (*Asc*), *Xbp1*, *Nlrp3* (cryopyrin), and *Par-2* in RAW 264.7. Finally, geniposide has been shown to suppress the levels of cytokines, H_2_O_2_, and NO in LPS-provoked RAW 264.7 through the calcium pathway.

## 2. Results

### 2.1. Effect of Geniposide on Cell Viability

**Figure 2:**
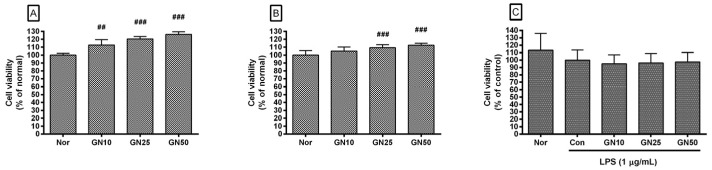
Effect of geniposide on cell viability after 24 h (**A**) and 48 h (**B**) treatment in RAW 264.7 as well as cell viability after 24 h treatment in lipopolysaccharide (LPS)-stimulated RAW 264.7 (**C**). Nor, normal group (media only); Con, control group (1 µg/mL of LPS alone). GN10, GN25, and GN50 indicate 10, 25, and 50 µM of geniposide, respectively. Values are the mean ± SD of three independent experiments (n = 9). Significant differences were examined using the Kruskal-Wallis test. ## *p* < 0.01 vs. Nor; ### *p* < 0.001 vs. Nor.

Exposure to geniposide at concentrations of 10, 25, and 50 µM did not decrease the viability of RAW 264.7 after 24 h and 48 h treatment. Cell viabilities in RAW 264.7 treated for 24 h with geniposide at concentrations of 10, 25, and 50 µM were 112.75 ± 6.78%, 120.37 ± 3.22%, and 126.31 ± 3.25% of the normal group (media only), respectively (Figure 2A); for 48 h treatment, cell viabilities were 104.87 ± 5.32%, 109.26 ± 3.75%, and 112.44 ± 2.45% of the normal group, respectively (Figure 2B). Geniposide did not show significant changes in the cell viability of RAW 264.7 stimulated with LPS (Figure 2C). Cell viabilities of RAW 264.7 treated with geniposide at concentrations of 10, 25, and 50 µM were 95.04 ± 12.01%, 96.17 ± 12.64%, and 97.53 ± 12.6% of the control group treated with LPS only, respectively. This finding indicates that geniposide does not reduce the production of inflammatory factors by causing cytotoxicity and consequently inhibiting the transcription of inflammation-related genes.

### 2.2. Effect of Geniposide on the Level of Hydrogen Peroxide, NO, and Ca^2+^

**Figure 3:**
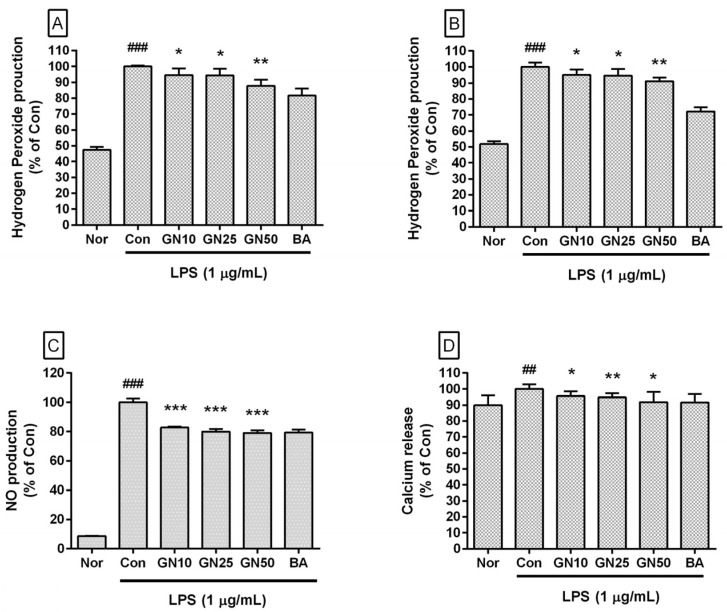
Effect of geniposide on hydrogen peroxide production for 24 h (**A**) and 48 h (**B**) treatment in lipopolysaccharide (LPS)-stimulated RAW 264.7 as well as NO production for 24 h treatment (**C**) and calcium release for 18 h treatment (**D**). Nor, normal group (media only); Con, control group (1 µg/mL of LPS alone). GN10, GN25, and GN50 indicate 10, 25, and 50 µM of geniposide, respectively. BA denotes baicalein (25 µM). Values are the mean ± SD of three independent experiments. Significant differences were examined using a one-way analysis of variance test followed by Tukey’s multiple comparison test. ## *p* < 0.01 vs. Nor; ### *p* < 0.001 vs. Nor; * *p* < 0.05 vs. Con; ** *p* < 0.01 vs. Con; *** *p* < 0.001 vs. Con.

Geniposide significantly inhibited the production of hydrogen peroxide in LPS-stimulated RAW 264.7 for 24 h and 48 h of treatment (Figure 3A,B). Geniposide significantly inhibited NO levels in LPS-stimulated RAW 264.7 (IC_50_:135.9 µM) (Figure 3C). Geniposide also significantly inhibited Ca^2+^ release in LPS-stimulated RAW 264.7 (IC_50_: 503.5 µM) (Figure 3D). Baicalein, a flavonoid, and aglycon hydrolyzed from baicalin, inhibited hydrogen peroxide generation, NO production, and Ca^2+^ release in LPS-stimulated RAW 264.7. Considering the inhibitory effect of baicalein, it is estimated that the inhibitory effect of geniposide on Ca^2+^ release in LPS-stimulated RAW 264.7 is also related to the anti-inflammatory effect of geniposide.

### 2.3. Effect of Geniposide on Cytokines Production

**Figure 4:**
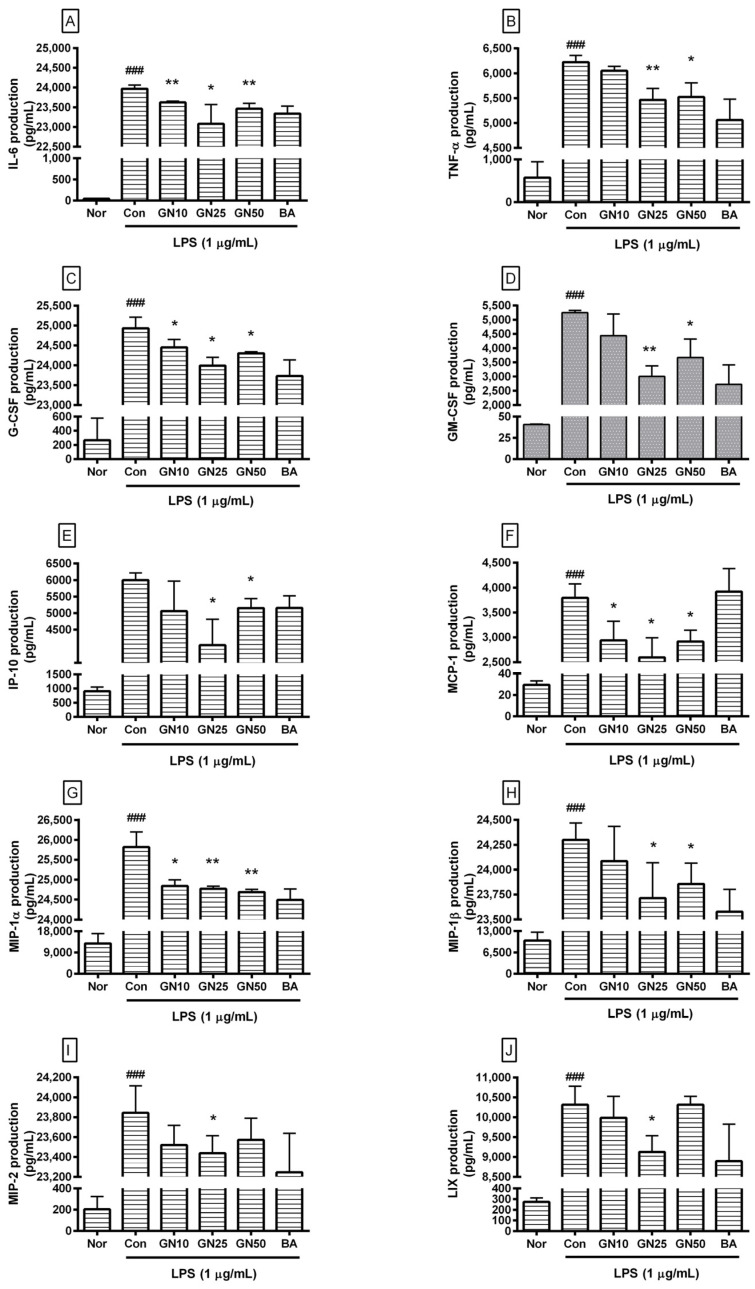
Effect of geniposide on the production of IL-6 (**A**), TNF-α (**B**), G-CSF (**C**), GM-CSF (**D**), IP-10 (**E**), MCP-1 (**F**), MIP-1α (**G**), MIP-1β (**H**), MIP-2 (**I**), and LIX (**J**) in LPS-stimulated RAW 264.7. Nor, normal group (media only); Con, control group (1 µg/mL of LPS alone). GN10, GN25, and GN50 indicate 10, 25, and 50 µM of geniposide, respectively. BA denotes baicalein (25 µM). Values are mean ± SD of three independent experiments. Significant differences were examined using a one-way analysis of variance test followed by Tukey’s multiple comparison test. ### *p* < 0.001 vs. Nor; * *p* < 0.05 vs. Con; ** *p* < 0.01 vs. Con.

Geniposide significantly decreased the production of IL-6, TNF-α, G-CSF, GM-CSF, IP-10, MCP-1, MIP-1α, MIP-1β, MIP-2, and LIX (CXCL5) in LPS-stimulated RAW 264.7 (Figure 4). However, the effect of geniposide at a concentration of 50 µM on the production of MIP-2 and LIX was not statistically significant.

Data revealed that geniposide exhibited IC50 values of 1454, 310.3, 1289, 65.55, 128.6, 925.8, 91.08, 846.2, 1949, and 2569 µM for IL-6, TNF-α, G-CSF, GM-CSF, IP-10, LIX, MCP-1, MIP-1α, MIP-1β, and MIP-2, respectively. Data suggest that geniposide exhibits anti-inflammatory effects in LPS-stimulated RAW 264.7 by reducing various cytokines, leading to amelioration of the hyper-inflammatory syndrome, known as cytokine storm, caused by endotoxemia. The already known inhibitory effect of baicalein on cytokine production could also be confirmed.

### 2.4. Effect of Geniposide on Inflammatory Target Genes Expressions

**Figure 5:**
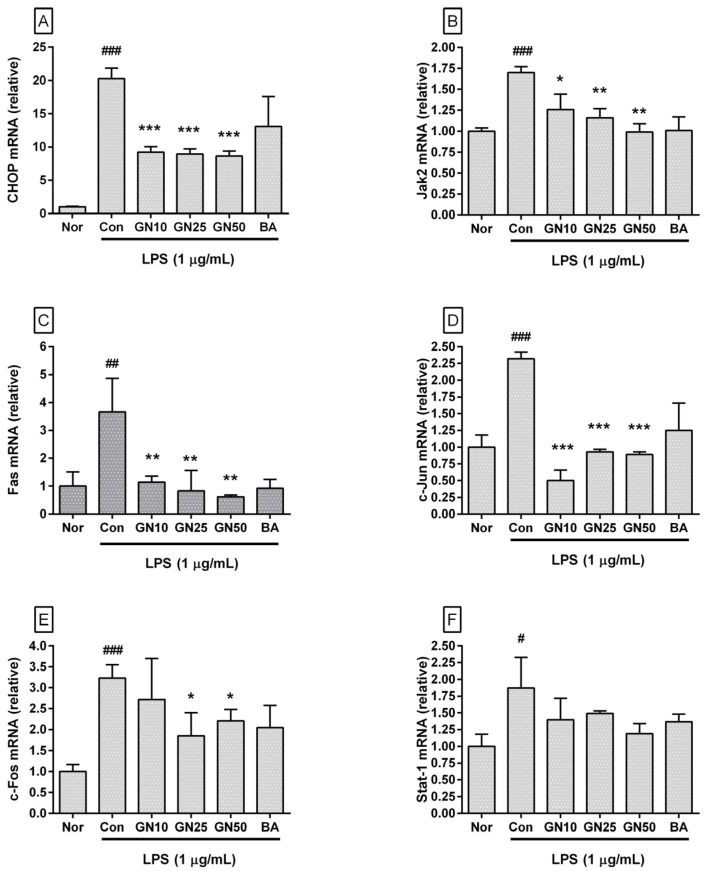
Effect of geniposide on the mRNA expression of *Chop* (**A**), *Jak2* (**B**), *Fas* (**C**), *c-Jun* (**D**), *c-Fos* (**E**), and *Stat-1* (**F**) in LPS-stimulated RAW 264.7. Nor, normal group (media only); Con, control group (1 µg/mL of LPS alone). GN10, GN25, and GN50 indicate 10, 25, and 50 µM of geniposide, respectively. BA denotes baicalein (25 µM). Values are mean ± SD of three independent experiments. Significant differences were examined using a one-way analysis of variance test followed by Tukey’s multiple comparison test. # *p* < 0.05 vs. Nor; ## *p* < 0.01 vs. Nor; ### *p* < 0.001 vs. Nor; * *p* < 0.05 vs. Con; ** *p* < 0.01 vs. Con; *** *p* < 0.001 vs. Con.

**Figure 6:**
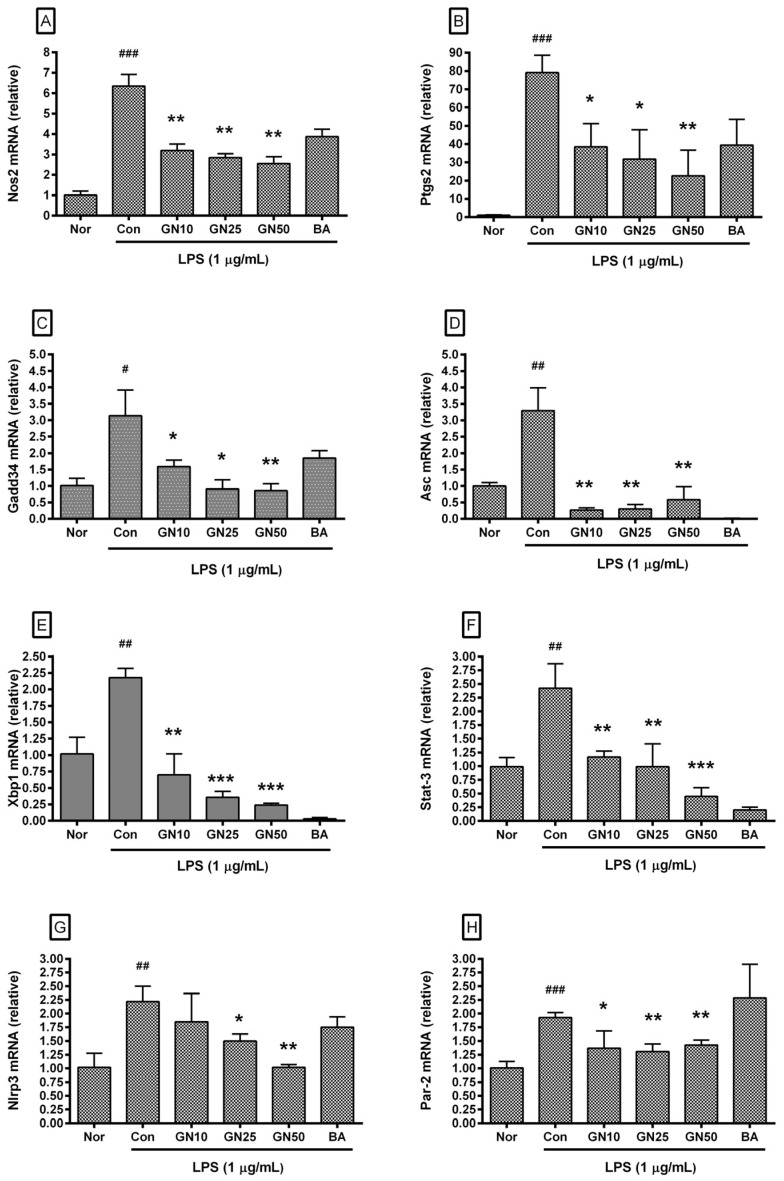
Effect of geniposide on the mRNA expression of *Nos2* (**A**), *Ptgs2* (**B**), *Gadd34* (**C**), *Asc* (**D**), *Xbp1* (**E**), *Stat-3* (**F**), *Nlrp3* (**G**), and *Par-2* (**H**) in LPS-stimulated RAW 264.7. Nor represents the normal group treated with medium only. Nor, normal group (media only); Con, control group (1 µg/mL of LPS alone). GN10, GN25, and GN50 indicate 10, 25, and 50 µM of geniposide, respectively. BA denotes baicalein (25 µM). Values are mean ± SD of three independent experiments. Significant differences were examined using a one-way analysis of variance test followed by Tukey’s multiple comparison test. # *p* < 0.05 vs. Nor; ## *p* < 0.01 vs. Nor; ### *p* < 0.001 vs. Nor; * *p* < 0.05 vs. Con; ** *p* < 0.01 vs. Con; *** *p* < 0.001 vs. Con.

Geniposide significantly affected the transcription of *Chop*, *Jak2*, *Fas*, *c-Jun*, *c-Fos*, *Stat-3*, *Nos2*, *Ptgs2*, *Gadd34*, *Asc*, *Xbp1*, *Nlrp3*, and *Par-2* genes (Figure 5 and Figure 6). However, statistical significance was not confirmed for the inhibitory effect on *Stat-1*. These data indicated that geniposide inhibited the production of inflammatory mediators via the ERS/CHOP-related pathway. The already known inhibitory effect of baicalein on the expression of ERS-related genes could also be confirmed.

### 2.5. Effect of Geniposide on P38 MAPK Phosphorylation

**Figure 7:**
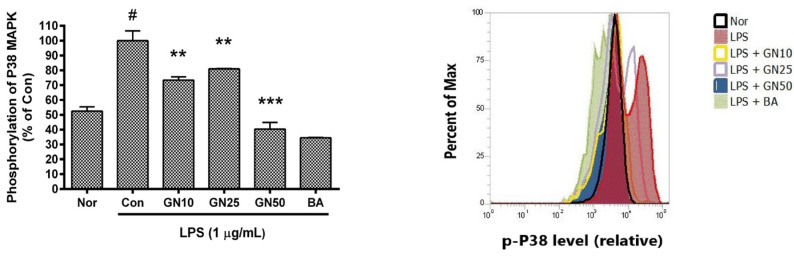
Effects of geniposide on the level of phosphorylated P38 MAPK in lipopolysaccharide (LPS)-stimulated RAW 264.7. The level of phosphorylated P38 MAPK was measured using Flow Cytometric Analysis. Values are the mean ± SD of three independent experiments. Significant differences were examined using a one-way analysis of variance test followed by Tukey’s multiple comparison test. Nor, normal group (media only); Con, control group (1 µg/mL of LPS alone); BA, baicalein (25 µM); p-P38, phosphorylated P38 MAPK. GN10, GN25, and GN50 indicate 10, 25, and 50 µM of geniposide, respectively. # *p* < 0.05 vs. Nor; *** *p* < 0.001 vs. Con; ** *p* < 0.01 vs. Con.

Geniposide meaningfully decreased the level of phosphorylated P38 MAPK in LPS-stimulated RAW 264.7 (Figure 7). These data indicate that geniposide exerts anti-inflammatory effects on LPS-provoked RAW 264.7 via the calcium pathway. The already known inhibitory effect of baicalein on P38 activation was also confirmed.

## 3. Discussion

Research on natural products for treating various inflammatory diseases continues [21]. Geniposide is a major constituent of the herb gardenia, which has been used to treat inflammatory disorders, fever, hypertension, edema, jaundice, and dysphoria for centuries [13,14,22]. Since geniposide is a major component of gardenia, if the inhibitory effect of geniposide on infectious inflammatory reactions is revealed in more detail, it will help develop anti-inflammatory substances using gardenia or develop new treatments to prevent worsening inflammatory diseases caused by infection.

Shi et al. reported that geniposide inhibited nitric oxide synthase in LPS-provoked macrophages, as well as cyclooxygenase-2 [14]. Interesting reports have been made about the anti-asthmatic properties of geniposide [23], anti-tumor activity of geniposide [24], anti-angiogenic activity of the herb gardenia [25], and anti-inflammatory activity of geniposide in oxygen/glucose-deprived rat microglial cells [26]. Liu et al. reported that geniposide inhibits IL-6 and IL-8 production in LPS-induced human umbilical vein endothelial cells by blocking P38 and ERK 1/2 signaling [27]. However, the complete mechanism underlying the bioactivity of geniposide remains unresolved.

LPS can stimulate immune cells like macrophages to secrete various inflammatory factors, including NO, cytokines, prostaglandins, and reactive oxygen species. Excessive production of inflammatory mediators in infectious diseases may have a serious outcome in patients. The production of large amounts of inflammatory factors from activated macrophages is associated with the exacerbation of sepsis [14,28]. Thus, LPS-provoked macrophages are regarded as being useful in the search for new anti-inflammatory agents.

Although many studies have evaluated the pharmacological activities of geniposide, its specific effects on cytokines secreted from LPS-provoked macrophages have not been fully reported until now. Therefore, the effects of geniposide on ERS-related cytokine production in LPS-provoked RAW 264.7 were investigated in this study.

Uncontrolled inflammation can develop into acute inflammatory diseases and further chronic inflammatory diseases such as diabetes [29]. Sepsis is a representative disease characterized by uncontrolled inflammation and a hyperinflammatory response (cytokine storm). Hu et al. have reported that sepsis is a systemic response that results from a harmful host response to infection [30].

Sun and Bhatia reported that MIP-1α, MCP-1, and MIP-2 are elevated after acute pancreatitis induced by cerulein in a mouse model. They also suggested that MIP-1α, MCP-1, and MIP-2 play a role in the pathogenesis of acute pancreatitis [31]. IL-6, an important factor in B-cell maturation and autoantibody production, induces articular and systemic symptoms of rheumatoid arthritis [32]. MCP-1 is hypersecreted in airway hyper-reactivity and chronic airway inflammatory diseases, such as asthma [33]. The exaggerated allergic inflammation with airway hyper-responsiveness can be accompanied by an increase in G-CSF and KC [34]. GM-CSF and IL-5 are clinically important in the pathophysiology of allergies and asthma [35]. During lung inflammation, levels of MCP-1, IP-10, G-CSF, and GM-CSF are increased in the bronchoalveolar fluid [36]. In the current study, geniposide at concentrations of 10, 25, and 50 μM meaningfully suppressed the production of IL-6, G-CSF, MCP-1, and MIP-1α in LPS-stimulated RAW 264.7 and geniposide at concentrations of 25 and 50 μM meaningfully suppressed levels of TNF-alpha, IP-10, GM-CSF, and MIP-1β. These experimental results indicate that various hyperinflammatory phenomena caused by endotoxins can be alleviated by geniposide. More research is needed to determine which inflammatory diseases geniposide will have a valid effect on.

**Figure 8:**
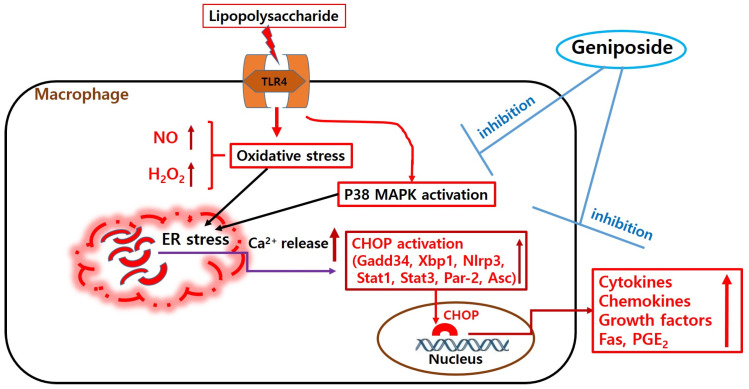
A diagram showing the effects of geniposide on lipopolysaccharide-provoked RAW 264.7 through the calcium pathway. Arrows mean increasing.

Interestingly, the current data showed that geniposide significantly inhibited the levels of intracellular Ca^2+^, NO, and hydrogen peroxide in LPS-provoked RAW 264.7; geniposide decreased significantly the transcription of *Chop*, *Jak2*, *Fas*, *c-Jun*, *c-Fos*, *Stat-3*, *Nos2*, *Ptgs2*, *Gadd34*, *Asc*, *Xbp1*, *Nlrp3*, and *Par-2* genes as well as P38 MAPK phosphorylation. LPS stimulation is well known to make RAW 264.7 produce cytokines and ROS via P38 MAPK activation [37]. Because ROS-mediated up-regulation of TACE activity in LPS-stimulated monocytes might progress via P38 MAPK activation [10], the inhibitory effects of geniposide on ROS production and expression of ERS-related genes in LPS-stimulated RAW 264.7 could be interpreted as geniposide inhibiting cytokine production, such as TNF-α and MCP-1, through regulation of ERS-related P38 signaling (Figure 8).

CHOP (GADD153) in stressed cells is activated by P38 MAPK [38]. LPS causes ERS with the overexpression of *Chop* [39]. During inflammation, a characteristic phenomenon occurs in which intracellular calcium concentration increases while ER calcium storage decreases [40]. CHOP-amplified cytoplasmic calcium is known to activate ROS generation via *Camk2a* activation [41]. ERS-induced calcium release might be related to *Camk2a* and Stat1 activation in macrophages [42]. In addition to CHOP, GADD34 [11,12], XBP1 [13], and PAR-2 [14] may also be upregulated through P38 MAPK phosphorylation in ERS reaction [11,12,13,14]. ASC and NLRP3 play an important role in inflammasome complex activation caused by infectious pathogens and in infection-induced macrophage activation [43]. Interestingly, the induction of ASC and NLRP3 inflammasomes plays an intermediate role in a series of inflammatory cascades from ERS to pyroptosis, which begins with ROS production and subsequently results in cellular injury [44]. In this study, it was not possible to determine whether XBP1 directly activates the transcription factor activity of CHOP. Since oxidative stress and ERS can cause each other, additional investigation is needed to determine whether geniposide suppresses ERS through the regulation of oxidative stress or whether it suppresses oxidative stress through the regulation of ERS. These results indicate that geniposide could modulate M1-type polarization in LPS-stimulated macrophages, which might be induced by ERS in bacterial infections [45]. Further studies are needed to verify the effects of geniposide on the conversion of M1 to M2 macrophages. Finally, geniposide significantly inhibited the levels of NO, ROS, IL-6, TNF-α, G-CSF, GM-CSF, IP-10, MCP-1, MIP-1α, and MIP-1β in LPS-stimulated RAW 264.7 via the calcium pathway. A shortfall of this study was the inability to use primary peritoneal or bone marrow-derived macrophages. Another shortcoming of this study is the inability to confirm the protein levels of CHOP, STAT-3, PTGS2, NOS2, GADD34, XBP1, PAR-2, Asc, and NLRP3, as well as the phosphorylation of STATs. It is also unfortunate that in this study, the concentration and treatment time of LPS that stimulates RAW 264.7 were not treated differently. Regrettably, the anti-inflammatory efficacy of geniposide could not be confirmed using animal test models due to the lack of research time and equipment. More research is needed to elucidate the medicinal benefits of geniposide in inflammatory disorders.

## 4. Materials and Methods

The experimental technique implemented in this study is based on previous reports [20,21], and detailed information is provided in the Supplementary Materials including Table S1 and Sections S1.1–S1.10.

### 4.1. Materials 

Chemicals for these experiments, including DMEM, were purchased from Thermo Fisher Scientific (Waltham, MA, USA). The treatment concentrations of geniposide (10~50 µM) were determined according to previous studies [18]. As in the previous study [20], baicalein, which is well known for its anti-inflammatory effects, was used as a positive control. Because the activation time of p38 MAPK in cells is due to stimulating factors, LPS and geniposide were administered simultaneously within a few hours.

### 4.2. Methods

#### 4.2.1. Cell Culture and Cell Viability

RAW 264.7 was obtained from the Korea Cell Line Bank (Seoul, Republic of Korea) and cultured in DMEM. In some cases, drug candidates may reduce the production of inflammatory factors, such as cytokines, by being toxic to macrophages; therefore, it is meaningful to investigate the effects of candidate substances on the cell viability of macrophages. Cell viability was measured using the MTT assay [20].

#### 4.2.2. Levels of H_2_O_2_, NO, and Ca^2+^ in Cells

Since ROS, such as H_2_O_2,_ play an important role in mediating and amplifying inflammatory responses and changing related immune cells, it is meaningful to investigate the effect on the generation of ROS in verifying the anti-inflammatory effect. The production of H_2_O_2_ was measured by the dihydrorhodamine (DHR) 123 assay [21]. Nitric oxide, one of the active nitrogen species, dilates blood vessels and activates local blood flow to help the aggregation of immune cells in the immune inflammatory response; therefore, increased nitric oxide production naturally leads to amplification of the inflammatory response. Therefore, most substances that exhibit anti-inflammatory effects often suppress the amount of nitric oxide produced by macrophages. The NO level was measured using the Griess reagent assay [20]. Increased release of intracellular calcium, especially from the endoplasmic reticulum, is an important trigger for triggering an inflammatory response along with ERS. Therefore, suppressing the increase in intracellular calcium release exhibits an anti-inflammatory effect that is involved in the ERS mechanism. Intracellular Ca^2+^ release was measured using Fluo-4 NW Calcium Assay Kits (Thermo Fisher Scientific, Waltham, MA, USA) [20].

#### 4.2.3. Levels of Inflammatory Cytokines 

With the recent development of advanced multiplex screening assays, it has become possible to simultaneously measure the amount of various cytokines in single-cell culture supernatants. In conducting experimental research, if the production of various cytokines can be measured simultaneously in the same cell culture supernatant, changes in the inflammatory response due to potential anti-inflammatory substances can be more accurately identified. The production of cytokines (IL-6, TNF-α, MIPs, G-CSF, etc.) was measured using the MILLIPLEX MAP Mouse Cytokine/Chemokine Magnetic Bead Panel kit (Millipore, Billerica, MA, USA) [20].

#### 4.2.4. Quantitative Real-Time RT-PCR for Inflammatory Genes

CHOP protein is a transcription factor that is activated in association with ERS and P38 MAPK activation and promotes the production of pro-inflammatory cytokines. Inflammation-related genes, such as *Fas* and *Stat*, are also associated with ERS and increased intracellular calcium release. GADD34, XBP1, and PAR-2 might be upregulated through P38 MAPK phosphorylation in the ERS reaction. The expression of *Nos2* is increased during inflammatory responses, resulting in increased production of nitric oxide. Increased expression of *Ptgs2* leads to increased production of prostaglandin E2 and promotes an inflammatory response. JAK2 cooperates with STAT1 and STAT3 to generate cellular signaling pathways and provides important clues to understanding various inflammatory diseases. The induction of ASC and NLRP3 inflammasomes plays an intermediate role in a series of inflammatory cascades from ERS to pyroptosis. c-Jun and c-Fos play important roles in regulating inflammation-related gene expression. The transcription of target genes such as *Chop*, *Jak2*, *Fas*, *c-Jun*, *c-Fos*, *Stat-1*, *Stat-3*, *Nos2*, *Ptgs2*, *Gadd34*, *Xbp1*, *Par-2*, *Nlrp3*, and *Asc* was evaluated using quantitative real-time RT-PCR [20].

#### 4.2.5. Phosphorylation of P38 MAPK

P38 MAPK is known to mediate inflammatory responses. It is activated by ROS, increases the production of NO, increases the production of pro-inflammatory cytokines such as IL-6, expands the intracellular response of ERS, and activates nuclear factor Kappa B, which causes inflammatory cascade and apoptosis. Flow cytometry was used to check phosphorylated P38 MAPK with an Attune NxT flow cytometer (Thermo Fisher Scientific) [20].

#### 4.2.6. Statistical Analysis

GraphPad Prism software (version 6.0; GraphPad Software, San Diego, CA, USA) was used for statistical analysis, including one-way analysis of variance.

## 5. Conclusions

Geniposide significantly inhibited levels of Ca^2+^, NO, H_2_O_2_, IL-6, TNF-α, G-CSF, GM-CSF, IP-10, MCP-1, MIP-1α, MIP-1β, and phosphorylated P38 MAPK as well as mRNA expression of *Chop*, *Jak2*, *Fas*, *c-Jun*, *c-Fos*, *Stat3*, *Nos2*, *Ptgs2*, *Gadd34*, *Asc*, *Xbp1*, *Nlrp3*, and *Par-2* in LPS-stimulated RAW 264.7, which means that the anti-inflammatory activity of geniposide in macrophages takes place through the calcium pathway.

## Data Availability

The data presented in this study are available on request from the corresponding author.

## Supplementary Materials

The following supporting information can be downloaded at: https://www.mdpi.com/article/10.3390/ijms25031728/s1.
