# Supplementary material for: Alleviative Effect of Geniposide on Lipopolysaccharide-Stimulated Macrophages via Calcium Pathway
Source: Int J Mol Sci. 2024 Jan 31;25(3):1728. doi: 10.3390/ijms25031728 (PMC10855527; doi:10.3390/ijms25031728)
Supplement: Supplementary file 1 [file ijms-25-01728-s001.zip › ijms-2818944-supplementary.pdf]

International Journal of
Molecular Sciences

MDPI

Article

Alleviative Effect of Geniposide on Lipopolysaccharide-Stimulated Macrophages via Calcium Pathway

Hyun-Ju Kim and Wansu Park *

Department of Pathology, College of Korean Medicine, Gachon University, Seongnam 13120, Republic of Korea

* Correspondence: pws98@gachon.ac.kr; Tel.: +82-31-750-8821

< Supplementary Material for Materials and Methods>

1. Materials and Methods

The following provides a detailed description of the materials and experimental methods used in this study.

1.1. Materials

Dulbecco's modified Eagle's medium (DMEM), FBS, penicillin, streptomycin, phosphate-buffered saline (1x), lipopolysaccharide (LPS) from Escherichia coli O55:B5 (Cat No. L2880; endotoxin levels of not less than 500,000 EU), geniposide, baicalein, and other cell culture reagents were purchased from Millipore (Billerica, MA, USA). Griess reagent assay kits and Fluo-4 calcium assay kits were purchased from Thermo Fisher Scientific (Waltham, MA, USA). Dihydrorhodamine 123 assay kits were purchased from Millipore. Multiplex cytokine assay kits were purchased from Millipore. Real time PCR kits were ordered from Bio-Rad (Hercules, CA, USA). Fixable Viability Dye eFluor 520 (eBioscience 65-0867-18), phospho-P38 MAPK (T180/Y182) Antibody (eBioscience 17-9078-42), and Mouse IgG2b kappa Isotype Control (eBioscience 12-4732-81) from Life Technologies Corporation (Carlsbad, CA, USA).

1.2. Aseptic preparation of geniposide

Geniposide (10~50 μM) was dissolved in phosphate buffer saline (1x) aseptically in clean bench, then filtered through a 0.22 μM syringe filter in order to avoid causing unnecessary infectious inflammatory reactions in cells.

1.3. Cell culture and cell viability

RAW 264.7 mouse macrophages were obtained from Korea Cell Line Bank (Seoul, Korea). RAW 264.7 mouse macrophages (passage number 4) were cultured with DMEM supplemented with 10% FBS containing 100 U/mL of penicillin and 100 μg/mL of streptomycin at 37 °C in a 5% CO_2 humidified incubator. Prior to the experimental assays, RAW 264.7 mouse macrophages were washed with phosphate buffer saline (1x). Briefly, RAW 264.7 mouse macrophages were incubated with geniposide for 24 h and 48 h in 96-well plates ($1x10^4$ cells/well) to verify the toxicity of geniposide. After incubation with geniposide, cell viability was confirmed with the tetrazolium-based colorimetric assay (a modified MTT assay). OD was determined at 540 nm with a Model 680 microplate reader (Bio-Rad, Hercules, CA, USA).

1.4. Quantification of NO Production in RAW 264.7 mouse macrophages

The NO concentration in culture medium was determined by the Griess reagent assay. Specifically, after incubating RAW 264.7 mouse macrophages ($1x10^4$ cells/well) with LPS and/or geniposide for 24 h, 100 μL of supernatant from each well was mixed with 100 μL Griess reagent in a 96-well plate. After 15 min of incubation at room temperature, the optical density was determined at 540 nm with a Model 680 microplate reader (Bio-Rad, Hercules, CA, USA). Baicalein (25 μM) was used as a positive control.

1.5. Ca^{2+} release in RAW 264.7 mouse macrophages

The Ca^{2+} release from RAW 264.7 mouse macrophages was measured using a Fluo-4 calcium assay kit (Thermo Fisher Scientific). RAW 264.7 mouse macrophages in 96-well plates ($1x10^5$ cells/well) were incubated with LPS and/or geniposide for 24 h at 37 °C. Next, the medium was removed, and the cells were incubated with 100 μL of the Fluo-4 dye loading solution for 30 min at 37 °C. After the incubation, the fluorescence intensity in each well was determined using

TRIAD LT spectrofluorometer with excitation and emission filters of 485 nm and 535 nm, respectively. Baicalein (25 μM) was used as a positive control.

1.6. Hydrogen peroxide production in RAW 264.7 mouse macrophages

Hydrogen peroxide production in RAW 264.7 mouse macrophages ($1x10^4$ cells/well) was measured by dihydrorhodamine 123 (DHR) assay. During the cellular production of reactive oxygen species, the nonfluorescent DHR was oxidized by H_2O_2 and irreversibly converted to the green fluorescent compound rhodamine 123 (R123). R123 was membrane-impermeable and accumulated in the cells. An aliquot of DHR (prepared to produce a concentration of 10 μM in each well) was added to each 96-well plate and pre-incubated for 30 min at 37 °C. Then, the medium was removed, and RAW 264.7 mouse macrophages were incubated with LPS and/or geniposide for 24 h and 48 h at 37 °C. After incubation, the fluorescence intensities of each well were analyzed by a spectrofluorometer (Dynex) with an excitation filter 485 nm and an emission filter 535 nm. Baicalein (25 μM) was used as a positive control.

1.7. Multiplex Cytokine Assay for cytokines production in RAW 264.7 mouse macrophages

Cytokines from RAW 264.7 mouse macrophages after 24 h of treatment were evaluated with MILLIPLEX MAP Mouse Cytokine/Chemokine Magnetic Bead Panel kits (Millipore) and a Bio-Plex 200 suspension array system (Bio-Rad, Hercules, CA, USA). Briefly, RAW 264.7 mouse macrophages were seeded in 96-well plates ($1x10^4$ cells/well) and treated with LPS and/or geniposide. After 24 h of treatment, the levels of the following cytokines in each well were analyzed: interleukin (IL)-6, tumor necrosis factor (TNF)-α, monocyte chemoattractant protein (MCP)-1, interferon inducible protein-10 (IP-10), granulocyte colony-stimulating factor (G-CSF), granulocyte macrophage colony-stimulating factor (GM-CSF), macrophage inflammatory proteins (MIP)-1α, MIP-1β, MIP-2, and lipopolysaccharide-induced CXC chemokine (LIX; CXCL5). Cytokines were measured using a Luminex assay based on xMAP technology using MILLIPLEX MAP Mouse Cytokine/Chemokine Magnetic Bead Panel kits and a Bio-Plex 200 suspension array system. The assay conducted in this experiment was designed for the multiplexed quantitative measurement of multiple cytokines in a single well, and as little as 25 μL of cell culture supernatant was used. The standard curves for each cytokine were generated using the kit-supplied reference cytokine samples. Briefly, the following procedure was performed: after pre-wetting the 96-well plate with Wash Buffer, the Wash Buffer was removed from each well using a Handheld Magnetic Separation Block (HMSB). Next, cell culture supernatants from each well were incubated with antibody-conjugated beads on a plate shaker for 2 h at room temperature. After incubation, well contents were gently removed with a HMSB, and the 96-well plate was washed twice. Then, 25 μL of Detection Antibodies was added to each well and incubated with agitation on a plate shaker for 1 h at room temperature. Subsequently, 25 μL Streptavidin–Phycoerythrin was added to each well containing the Detection Antibodies and incubated for 30 min with agitation on a plate shaker at room temperature. After incubation, the well contents were gently removed and washed 2 times using a HMSB. Then, 150 μL of Sheath Fluid was added to all wells, and the beads bound to each cytokine were analyzed with a Bio-Plex 200 instrument (Bio-Rad). The raw data (fluorescence intensity) were analyzed using Bio-Plex Manager software (Bio-Rad). Baicalein (25 μM) was used as a positive control.

1.8. Quantitative Real time PCR for mRNA Expression in RAW 264.7 mouse macrophages

Total RNA of RAW 264.7 mouse macrophages ($1x10^6$ cells/well) was isolated using NucleoSpin RNA kit (Macherey-Nagel, Duren, Germany). Then, cDNA of the RNA samples was synthesized using iScript cDNA Synthesis kit (Bio-Rad) and checked with an Experion Automatic Electrophoresis System (Bio-Rad). The transcription levels of C/EBP homologous protein (*Chop*), Janus kinase 2 (*Jak2*), First apoptosis signal receptor (*Fas*), Nitric oxide synthase 2 (*Nos2*), Prostaglandin-endoperoxide synthase 2 (*Ptgs2*), Signal Transducer and Activator of Transcription 1 (*Stat1*), *Stat3*, *c-Jun*, *c-Fos*, NLR family pyrin domain containing 3 (*Nlrp3)*, X-box binding protein 1 (*Xbp1*), growth arrest and DNA damage-inducible gene 34 (*Gadd34*), Apoptosis-associated speck-like protein containing a caspase recruitment do-main (*Asc*), protease-activated receptor-2 (*Par-2*), and *β-Actin* (internal control) were evaluated with Quantitative real time PCR using Bio-Rad CFX 96 (Bio-Rad). The details of the step-by-step experiment are as follows. Baicalein (25 μM) was used as a positive control.

1.8.1. Isolation of RNA

RAW 264.7 mouse macrophages were incubated with LPS and/or geniposide for 18 h in 6-well plates ($1x10^6$ cells/well). Baicalein (25 μM), a well-known anti-inflammatory flavonoid, was used as a positive control. After 18 h of incubation, the total RNA in each well was isolated using NucleoSpin RNA kit (Macherey-Nagel, Duren, Germany). Briefly, 350 μL Lysis Buffer RA1 and 3.5 μL β-mercaptoethanol were added to the cell pellet and vigorously vortexed to lyse cells.

Lysate was cleared by filtration using a NucleoSpin® Filter, then 350 μL ethanol (70%) was added, and mixed by vortexing. The lysate was loaded into the NucleoSpin® RNA Column, and 350 μL Membrane Desalting Buffer was added and centrifuged. 95 μL DNase reaction mixture was applied directly to the center of the silica membrane of the column, followed by incubation at room temperature for 15 min. The samples were washed with Wash Buffer RA2 and Wash Buffer RA3, and silica membrane was dried. RNA was eluted in 60 μL RNase-free water and centrifuged.

1.8.2. Determination of RNA concentration

RNA concentration was measured using Experion RNA StdSens Analysis kit (Bio-Rad) with the Experion Automatic Electrophoresis System (Bio-Rad). First, the electrodes were cleaned using a cleaning chip filled with 900 μL DEPC-treated water. Then, the Gel-Stain solution was prepared, 9 μL of the solution was added into labeled wells, and the chip was primed. Samples and RNA ladder were loaded into the chip, which was vortexed using the Experion vortex station for 1 min. Finally, the chip was loaded into the electrophoresis platform and the RNA StdSens Analysis program was run.

1.8.3. cDNA synthesis

cDNA of the RNA samples was produced using iScript cDNA Synthesis kit (Bio-Rad). Briefly, 20 μL complete reaction mixes were prepared using 5x iScript Reaction Mix (4 μL), iScript Reverse Transcriptase (1 μL), Nuclease-free water (variable), and RNA template (variable, 1 μg total RNA). The reaction mix (20 μL) was incubated in a thermal cycler (C1000 Thermal Cycler, Bio-Rad) according to the manufacturer's protocol (priming at 25°C for 5 min, reverse transcription at 46°C for 20 min, and RT inactivation at 95°C for 1 min).

1.8.4. Quantitative Real time PCR analysis

Gene expression was measured using quantitative polymerase chain reaction with iQ SYBR Green Supermix (Bio-Rad) using the CFX96 Real-Time PCR Detection System (Bio-Rad). Briefly, a master mix was prepared for all reactions by adding iQ SYBR Green Supermix and Forward/Reverse primers for each target gene. This master mix was thoroughly mixed to ensure homogeneity, and 7 μL was dispensed into the wells of a qPCR plate. Next, 3 μL of cDNA was added to each well; any air bubbles in the vessel bottom were removed, and the PCR plate was loaded into the real time PCR instrument. PCR was performed using the following protocol: denaturation of DNA at 95°C for 3 min, followed by 40 cycles of 95°C for 10 sec and 55°C for 30 sec. The $2^{-\Delta\Delta Ct}$ cycle threshold method was used to normalize the relative mRNA expression levels to the internal control, *β-actin*. Table 1 lists the primers used in this assay.

Table S1. Primers used in quantitative real time PCR

Name[1]	Forward Primer (5′–3′)	Reverse Primer (5′–3′)
Chop	CCACCACACCTGAAAGCAG	TCCTCATACCAGGCTTCCA
Jak2	TTGGTTTTGAATTATGGTGTCTGT	TCCAAATTTTACAAATTCTTGAACC
Fas	CGCTGTTTTCCCTTGCTG	CCTTGAGTATGAACTCTTAACTGTGAG
Nos2	TGGAGGTTCTGGATGAGAGC	AATGTCCAGGAAGTAGGTGAGG
Ptgs2	TCAAACAGTTTCTCTACAACAACTCC	ACATTTCTTCCCCCAGCAA
Stat1	TGAGATGTCCCGGATAGTGG	CGCCAGAGAGAAATTCGTGT
Stat3	GTCTGCAGAGT TCAAGCACCT	TCCTCAGTCACGATCAAGGAG
c-Jun	ACTGGGTTGCGACCTGAC	CAATAGGCCGCTGCTCTC
c-Fos	AGAGCGGGAATGGTGAAGA	TCTTCCTCTTCAGGAGATAGCTG
Nlrp3	GGGAGACCGTGAGGAAAGGA	CCAAAGAGGAATCGGACAACAAA
Xbp1	GAGTCCGCAGCAGGTG	GTGTCAGAGTCCATGGGA
Gadd34	CGCCGCGTCAGGGTATAA	TGACTCAATCTGCGCCAACA
Asc	GTGTTTACTCTCTGGGATGTTTTTG	GTCTGTGGAATTTAGGTGTTGGA
Par-2	CACCGGGACGCAACAACAG	GTAGACGACCGGAAGAAAGAC
β-Actin	CTAAGGCCAACCGTGAAAAG	ACCAGAGGCATACAGGGACA

[1]Primer names: C/EBP homologous protein (*Chop*), Janus kinase 2 (*Jak2*), First apoptosis signal receptor (*Fas*), Nitric oxide synthase 2 (*Nos2*), Prostaglandin-endoperoxide synthase 2 (*Ptgs2*), Signal Transducer and Activator of Transcription 1 (*Stat1*), *Stat3*, *c-Jun*, *c-Fos*, NLR family pyrin domain containing 3 (*Nlrp3*), X-box binding protein 1 (*Xbp1*), growth arrest and

DNA damage-inducible gene 34 (*Gadd34*), Apoptosis-associated speck-like protein containing a caspase recruitment domain (*Asc*), protease-activated receptor-2 (*Par-2*), and *β-Actin*.

1.9. Flow Cytometric Analysis for the level of Phosphorylated P38 MAPK

RAW 264.7 mouse macrophages were incubated with LPS and/or geniposide for 18 h in 6-well plates (3 x 10^5 cells/well), and the levels of phosphorylated P38 MAPK in RAW 264.7 were evaluated via flow cytometry using an Attune NxT flow cytometer (Thermo Fisher Scientific). Briefly, after 18 h of treatment, cells were stained with Fixable Viability Dye eFluor 520 (eBioscience 65-0867-18), phospho-P38 MAPK (T180/Y182) Antibody (eBioscience 17-9078-42), and Mouse IgG2b kappa Isotype Control (eBioscience 12-4732-81) according to the manufacturer's protocol. Fixable Viability Dye eFluor 520 was used to irreversibly label dead cells prior to cryopreservation, fixation, and/or permeabilization procedures. Cells were fixed with Fix Buffer (Thermo Fisher Scientific), permeabilized with Perm Buffer (Thermo Fisher Scientific), and stained with fluorescent-labeled antibodies. The stained cells were analyzed on the Attune NxT flow cytometer (Thermo Fisher Scientific). A serial gating strategy used forward scatter versus side scatter plots, forward scatter versus viability stain plots, and the target antibody expression plots. Unstained cells were used as the negative controls for gating. The data were obtained from the mean fluorescent intensities of the samples. Details regarding startup, proper calibration, and operation of the Attune can be found in the Attune User Guide (https://assets.thermofisher.com/TFS-Assets/LSG/manuals/100024235_AttuneNxT_HW_UG.pdf, accessed on 10 September 2023). Mouse IgG2b kappa Isotype Control was used to confirm the specificity of phospho-P38 MAPK Antibody. Data were obtained from the mean fluorescent intensities of samples. For analysis of raw data, Attune NxT software (Thermo Fisher Scientific) was used. Baicalein (25 μM) was used as a positive control.

1.10. Statistical analyses

Data are representative of three independent experiments, and the values are expressed in means ± standard deviation (SD). After testing for the normality of data, significant differences were examined using one-way analysis of variance test followed by Tukey's multiple comparison test or Kruskal-Wallis test, as appropriate. The program GraphPad Prism 6.0 software (GraphPad Software, San Diego, CA, USA) was used for statistics. A p-value < 0.05 was considered statistically.
